# Severe acute malnutrition morphological patterns in children under five

**DOI:** 10.1038/s41598-021-82727-x

**Published:** 2021-02-19

**Authors:** Laura Medialdea, Barry Bogin, Mbeugue Thiam, Antonio Vargas, María D. Marrodán, Nicole I. Dossou

**Affiliations:** 1 Technical Department, Action Against Hunger (AAH) Foundation, Madrid, Spain; 2 Laboratorio de Poblaciones del Pasado (LAPP), Departamento de Biología, Universidad Autónoma de Madrid (UAM), Madrid, Spain; 3 UCSD/Salk Center for Academic Research and Training in Anthropogeny (CARTA), University of California San Diego, San Diego, USA; 4 School of Sport, Exercise & Health Sciences, Loughborough University, Loughborough, UK; 5 Laboratoire de Recherche en Nutrition et Alimentation Humaine (LARNAH), Département de Biologie Animale, Université Cheikh Anta Diop de Dakar, Dakar, Senegal; 6 Grupo de Investigación EPINUT, Facultad de Medicina, Departamento de Biodiversidad, Ecología y Evolución, Universidad Complutense de Madrid (UCM), Madrid, Spain

**Keywords:** Developmental biology, Anatomy, Diseases, Mathematics and computing

## Abstract

Current methods for infant and child nutritional assessment rely on anthropometric measurements, whose implementation faces technical challenges in low- and middle-income countries. Anthropometry is also limited to linear measurements, ignoring important body shape information related to health. This work proposes the use of 2D geometric morphometric techniques applied to a sample of Senegalese participants aged 6–59 months with an optimal nutritional condition or with severe acute malnutrition to address morphometric variations due to nutritional status. Significant differences in shape and size body changes were described according to nutritional status, resulting age, sex and allometric effect crucial factors to establish nutritional morphological patterns. The constructed discriminant functions exhibited the best classification rates in the left arm. A landmark-based template registering body shape could be useful to both assess acute malnutrition and better understand the morphological patterns that nutritional status promotes in children during their first 5 years of growth and development.

## Introduction

Nutrition is a crucial factor for optimum child growth and development, especially throughout the first 1000 days of life, but also beyond. Globally, child undernutrition is a public health problem with major consequences for child survival, impairing physical and cognitive development of children, increasing their risk of death from infectious diseases and damaging the economic productivity of individuals and societies^1^. Geographically, 70–80% of undernourished children across the world live in low- and middle-income countries (LMIC)^2^, including Senegal, where the present study has been carried out.

Acute malnutrition affects 1 in 12 children worldwide (52 million) and contributes to more than 50% of all infant and child under-5-year-old deaths each year and 11% of the total global disability-adjusted-life-years worldwide^3^. Its most critical and often lethal condition, called Severe Acute Malnutrition (SAM), is a major cause of infant and child mortality^4^. Infants and children under 5 years old with SAM are nine times more likely to die than healthier children under 5 years of age^3^. With an alarming figure of one million deaths each year in the 5-year-old age range, SAM is among the deadliest forms of malnutrition in LMIC^1,3^.

Since SAM is an indicator of an emergency that requires urgent action^3^, both identification and treatment of all children with any of the current definitions of SAM mandated by the World Health Organization (WHO) is a public health priority^5^. The WHO defines SAM using three independent criteria ^6,7^: (1) weight-for-height/length z-score (WHZ) <  − 3 standard deviations (SD), (2) an absolute mid-upper-arm circumference (MUAC) of < 115 mm, or (3) the presence of nutritional bilateral oedema. According to WHZ, 500,000 to one million children die annually suffering from SAM, but if the mortality figures identified through the other two WHO definitions are included, the death toll may rise considerably^1,8^. Consequently, early diagnosis of SAM is critical in the management and reduction of children mortality^3,5^.

MUAC and WHZ have been shown to reliably predict outcome in severely malnourished children and are widely used to identify those at greater risk of death^8,9^. However, these indicators are based on indirect measurements of body composition and their interpretation and validity have been challenged^10,11^. Furthermore, anthropometric measurements in infants and children under 3 years old are less reliable, with respect to adults, as they are smaller and minor measuring differences may change significantly their nutritional assessment^12^. This circumstance adds to the difficulty of taking accurate and reliable measurements. Direct body measurements taken during an examination are time-consuming and need to follow rigorous protocols, which also require well-trained operators to minimize measurement errors^10,13^. Furthermore, the collaboration of the participants is essential, since they have to remain patiently still and follow the examiners instructions^14–16^. These demands become even more arduous when the subjects are under five years old. Therefore, the conception and development of new methods to study nutritional status in infancy and early childhood is relevant^17^.

In this respect, Geometric Morphometric (GM) methods can contribute to generate knowledge on how body shape changes with respect to nutritional status variations, since GM methods provide an effective set of tools to sample and analyze shape phenotypes via the evaluation of two or three-dimensional Cartesian coordinates of landmarks^18^. Furthermore, the relative position of landmarks used to register target shapes allows the gathering of geometric information. Such generated data enables the visualization of shape changes associated with landmark displacement across the shape space as well as the use of multivariate statistical analysis to quantify the magnitude of such changes. The application of GM to study human body shape, together with algorithms applied to body images, may empower anthropometric and nutritional applications through time-saving on data acquisition and enhancements in the reliability of measurements. Moreover, since target shape (infant and child bodies in our case) is preserved through the geometry of landmarks configurations, the association between shape and other measures can also be addressed.

Up to date, landmark-based models have often been used to define body dimensions as well as the anatomical correspondence between two different body images^13,14^ or to study human body modelling and its applications^16,19–22^. In a recent study, Medialdea et al.^17^ applied GM techniques to address ontogeny effects in child body shape, illustrating the potentiality of such methods to help understand the variations in shape and size of human body during development and growth.

It is estimated that more than half a million infant and child deaths caused by undernutrition annually could be prevented if the ability to screen and identify severely malnourished children at greater risk for death was improved as the treatment of these children could be started earlier and more vigorously to avert death^5^. Therefore, the aim of this study was, complementarily to the research line opened in a previous work^17^, that is, to assess body shape changes that occur between 6 and 59 months of age associated with SAM by means of geometric morphometric techniques applied to images of the body in anterior view in order to evaluate this new methodology to asses undernutrition.

## Results

### Morphometrical variation of infants and children under 5 years old

To examine infant and child body shape and size variation with respect to age group, sex and nutritional status, a Procrustes ANOVA was applied to the whole sample both before and after performing the allometric correction to Procrustes coordinates. A multivariate regression of shape on size explained 34.9% of shape variance (SSp = 0.243; p < 0.0001) and the resulting residuals were analyzed as shape variables with minimized relative size effect (Supplementary Table S1). Significant differences were found in shape variables among age, nutritional status and sex groups, both before and after reducing allometric effect (p < 0.0001), while significant differences for size (p < 0.0001) were found among age groups and nutritional status before minimizing the size effect and only for nutritional status once the size effect had been removed (Table 1). Data were also analyzed after subdividing the dataset by age group, sex and nutritional status, evaluating the effect of the remaining two factors for each case (Supplementary Table S2). A significant ontogenetic effect was found (*p* < 0.0001) for shape variables in all age and nutritional status groups before executing allometric correction, but only for infants and children with an optimal nutritional condition (ONC) and for males after implementing allometric correction. Significant differences were also found for the size component (centroid size, CS) among nutritional status and age groups in both coordinate models (*p* < 0.0001). Shape differences regarding nutritional status were statistically significant for all age and sex groups irrespectively of the size effect. Regarding the size component, they were only identified as significant in sex groups and < 24 months-olds before correcting for allometry. Finally, significant sexual dimorphism was reported in shape for all groups in both coordinate models with the sole exception of ONC infants and children in the allometry-minimized model.

**Table 1:** Ontogenetic, sexual and nutritional effects on children body shape and size.

Procrustes ANOVA	Effect	Shape					Size				
		SS	MS	df	F	p-value	SS	MS	df	F	p-value
Before Size correction	Age group	2.163E−01	7.618E−04	284	118.35*	**< 0.0001**	4.708E+07	4.708E+07	1	648.35*	**< 0.0001**
	Sex	4.355E−03	1.533E−05	284	2.38*	**< 0.0001**	7.729E+03	7.729E+03	1	0.11	0.7445
	Nutritional Status	4.355E−02	1.533E−04	284	23.82*	**< 0.0001**	1.999E+06	1.999E+06	1	27.53*	**< 0.0001**
After size correction	Age group	4.238E−03	1.492E−05	284	2.54*	**< 0.0001**	< 0.001E−4	< 0.001E−4	1	0.00	0.9764
	Sex	4.938E−03	1.739E−05	284	2.96*	**< 0.0001**	< 0.001E−4	< 0.001E−4	1	0.13	0.7201
	Nutritional status	4.685E−02	1.650E−04	284	28.05*	**< 0.0001**	1.700E−05	1.700E−05	1	45.22*	**< 0.0001**

Procrustes ANOVA test for shape (Procrustes coordinates) and size (log centroid size) in the whole sample by means of age groups, sex, and nutritional status.

Procrustes sums of squares (SS). Procrustes mean squares (MS). degrees of freedom (df). Goodall's F statistic (F). (*) Significance level: p < 0.0001.

**Figure 1:**
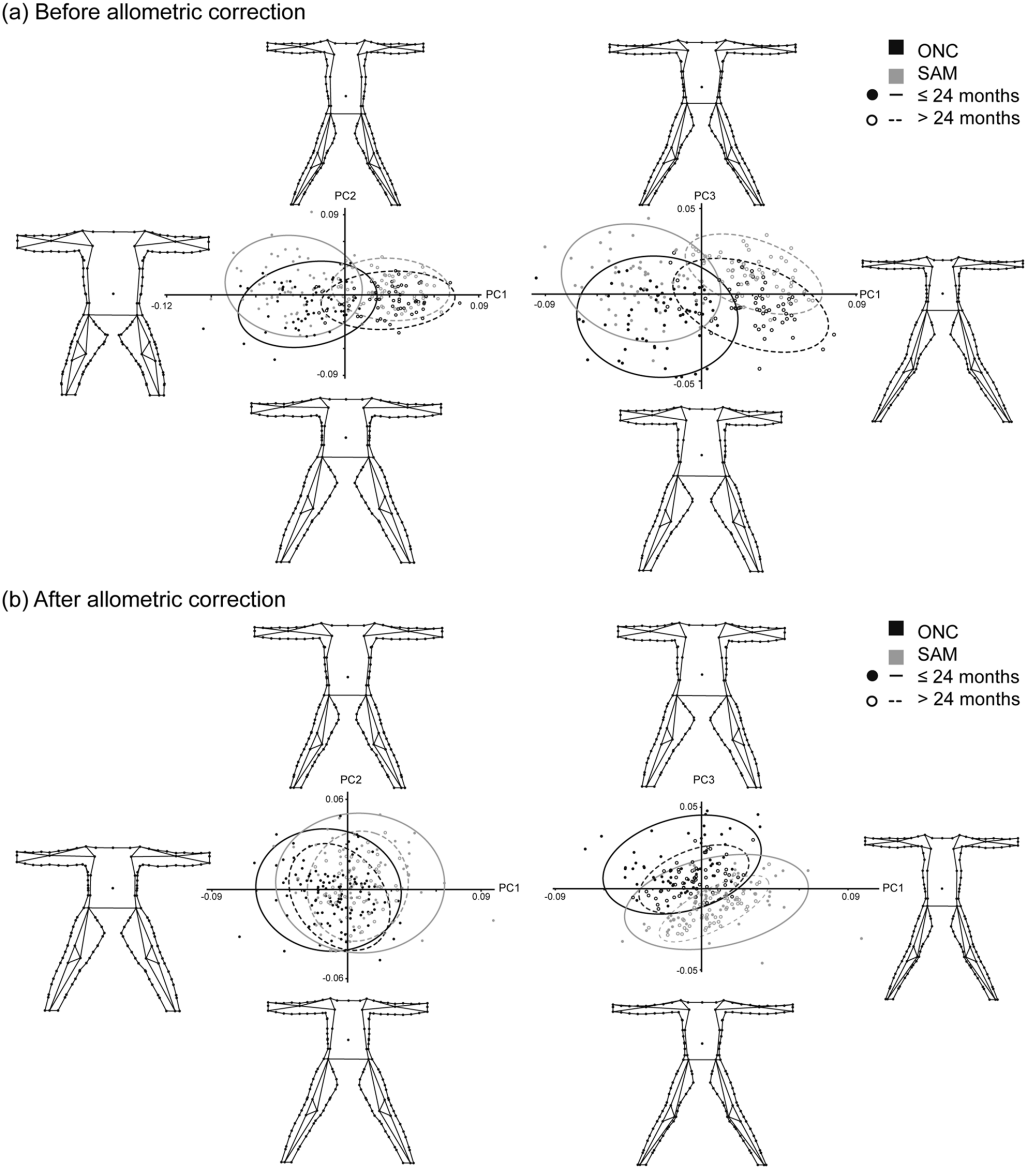
Shape variation of body shape in anterior view of infants and children aged 6–59 months. Principal component analysis both considering (**a**) and correcting for (**b**) allometric effect, showing main shape changes along PCs1-3. Plots of PC1 vs PC2 (left) and PC1 vs PC3 (right) exhibit the distribution of the sample according to their nutritional status, optimal nutritional condition (ONC) as black ellipses and dots and severe acute malnutrition (SAM) as grey ellipses and dots; together with age groups, under (filled dots) and over 24 months (unfilled dots).

A principal component analysis (PCA) was used to explore major body shape changes. A total of 239 principal components (PCs) were extracted from the analysis before carrying out the size correction, where PC1 explained 45.7% of the total variance, PC2 16.9% and PC3 10.1%, accounting all together for 72.8%. Negative forms for PC1 showed an overall plumped shape, with limbs thicker than average shape, longer arms and shorter legs, together with a broader and rounded trunk (Fig. 1a). In contrast, positive values for PC1 represent thinner limbs and trunk, resulting in trunk and arms reduced in amplitude. The trunk is displaced medially and arms downwards with respect to the mean shape. Legs appeared longer than the mean shape with a remarkable medial-proximal curvature. PC2 depicted the position and thickness of legs and arms, together with the variability in the dimensions of the trunk (Fig. 1a). Thus, positive forms showed thin and short legs linked to narrow hips, also exhibiting a long and slim trunk, with the umbilicus inferiorly displaced with respect to the average shape. Consequently, the arms were displaced upwards and presented a thin and short constitution. In contrast, negative forms represented thicker legs inserted in a wider hip, superiorly displaced, providing a higher length to the inferior limbs. The general morphometry of the trunk resulted in a reduced but broader structure with respect to the mean configuration, with the umbilicus superiorly displaced. The arms showed longer and thicker proportions. Finally, PC3 represents a general gain (negative forms) or loss (positive forms) in the overall thickening of the shape of the infant or child body, highlighting longer arms and legs in the thinnest forms and an expanded trunk in the thickest ones (Fig. 1a). When PC1 was plotted against PC2 and PC3, considering age group and nutritional status factors, PC1 represented shape variation among age groups while PC2 and PC3 more related to morphometrical variations between nutritional status groups (Fig. 1a). Sexual dimorphism was not evident in any of the three PCs since the shape of both girls and boys overlapped considerably in the morphospace depicted by the different PC combinations.

Once the size effect was minimized, the three first PC accounted for 59.0% of the total explained variance. PC1 (26.2% of the total variance) showed a slimming of the general body shape from negative to positive forms, together with an enlargement of the trunk while both inferior and superior limbs got shorter longitudinally (Fig. 1b). PC2 (19.0%) described the enlargement of superior limbs and trunk towards the positive values of the axis opposed to the most appreciable development of the inferior limbs in length and distally displaced depicted by negative values (Fig. 1b). Finally, PC3 (13.8%) defined general thickened shape in positive forms, with shorter legs and arms and a long trunk (Fig. 1b). Negative forms represented narrower body shapes with arms and legs longitudinally developed while the trunk appeared reduced. PC plotting showed different correspondence with age, sex and nutritional status (Fig. 1b). A great overlap was observed between girls and boys, and children under and over 24 months of age, not allowing the association of sex effect or ontogeny to any of the three PCs. However, nutritional status was strongly represented in PC1 and PC3.

### Nutritional status determination by means of shape

A discriminant function analysis (DFA) was used to evaluate the capability of landmark-based data to assess nutritional status. The first step of the analysis was to assess the whole-body configuration considering allometric effects, together with age and sex categories. The Hotelling *t*^2^ test was used to assess between-means differences and revealed significant differences (p < 0.0001) between ONC and SAM children, before carrying out the correction for allometry, but no significant differences (p > 0.05) after reducing such effect. (Table 2). Furthermore, significant differences (p < 0.0001) between the two nutritional statuses were described when age or sex groups were considered, regardless of the allometric effect in the analysis. When both factors were considered together, significant differences (p < 0.0001) between ONC and SAM children were found for any age and sex group except for girls under 24 months before minimizing the size effect. DFA precision ranged between 50.8% and 98.3% along the different datasets analyzed after performing a cross-validation (CV). The lowest correct classification was registered when the whole sample was considered (50.8–55.00%), increasing to values between 70 and 80% in girls and boys analyzed separately, and in girls and boys under 24 months, all regardless of the allometric effect, as well as the whole subsample of infants under 24 months before removing the size effect. The best classification accuracies (86.7–98.3%) corresponded to participants over 24 months and subsamples of girls and boys over 24 months before and after correcting for allometric effect together with infants under 24 months after correcting for allometry.

**Table 2:** Ontogenetic and sexual effect on nutritional status determination in the whole body.

Sample	Allometric effect	Levels	n	PrD	t^2^	p-value	ONC (%)	SAM (%)	Total (%)	Sp (%)	Se (%)
Global sample	Before size correction	Total	240	0.027	36,817.485*	**< 0.0001**	53.33	56.67	55.00	55.17	54.84
	After size correction	Total	240	0.028	6124.607	0.178	50.83	50.83	50.83	50.83	50.83

Factors	Allometric effect	Levels	n	PrD	t^2^	p-value	ONC (%)	SAM (%)	Total (%)	Sp (%)	Se (%)
Age group	Before size correction	< 24 months	120	0.031	2702.893*	**< 0.0001**	75.00	85.00	80.00	83.33	72.27
		> 24 months	120	0.025	4361.114*	**< 0.0001**	86.67	93.33	90.00	92.86	87.50
	After size correction	< 24 months	120	0.032	2624.544*	**< 0.0001**	83.33	95.00	89.17	94.34	85.07
		> 24 months	120	0.025	6732.561*	**< 0.0001**	91.67	93.33	92.50	93.22	91.80
Sex	Before size correction	Female	120	0.027	2248.524*	**< 0.0001**	75.00	83.33	79.17	81.82	76.92
		Male	120	0.028	2432.210*	**< 0.0001**	75.00	85.00	80.00	83.64	78.46
	After size correction	Female	120	0.029	2406.138*	**< 0.0001**	71.67	78.33	75.00	76.79	73.44
		Male	120	0.027	2015.156*	**< 0.0001**	75.00	75.00	75.00	75.00	75.00
Age group and sex	Before size correction	Female < 24 months	60	0.029	631.440	0.2310	70.00	73.33	71.67	72.41	70.97
		Female > 24 months	60	0.029	1038.609*	**< 0.0001**	90.00	96.67	93.33	96.43	90.63
		Male < 24 months	60	0.035	431.509*	**0.0020**	70.00	70.00	70.00	70.00	70.00
		Male > 24 months	60	0.023	801.654	**< 0.0001**	76.67	96.67	86.67	95.83	80.56
	After size correction	Female < 24 months	60	0.033	796.312*	**0.0080**	70.00	70.00	70.00	70.00	70.00
		Female > 24 months	60	0.028	1756.579*	**< 0.0001**	96.67	100.00	98.33	100.00	96.77
		Male < 24 months	60	0.033	508.972*	**< 0.0001**	70.00	73.33	71.67	72.41	70.97
		Male > 24 months	60	0.023	939.305*	**< 0.0001**	83.33	96.67	90.00	96.15	85.29

Discriminant Function Analysis (DFA) cross-validated (CV) results for nutritional status determination in the whole body of children considering age groups and sex independent and combined effect.

Procrustes distance means difference (PrD), Hotelling t2 statistic (t2). (*) Significance level: p < 0.01, after 10,000 permutation runs. Optimal nutritional condition (ONC), severe acute malnutrition (SAM). Specificity (Sp) and sensibility (Se) estimated for SAM.

**Figure 2:**
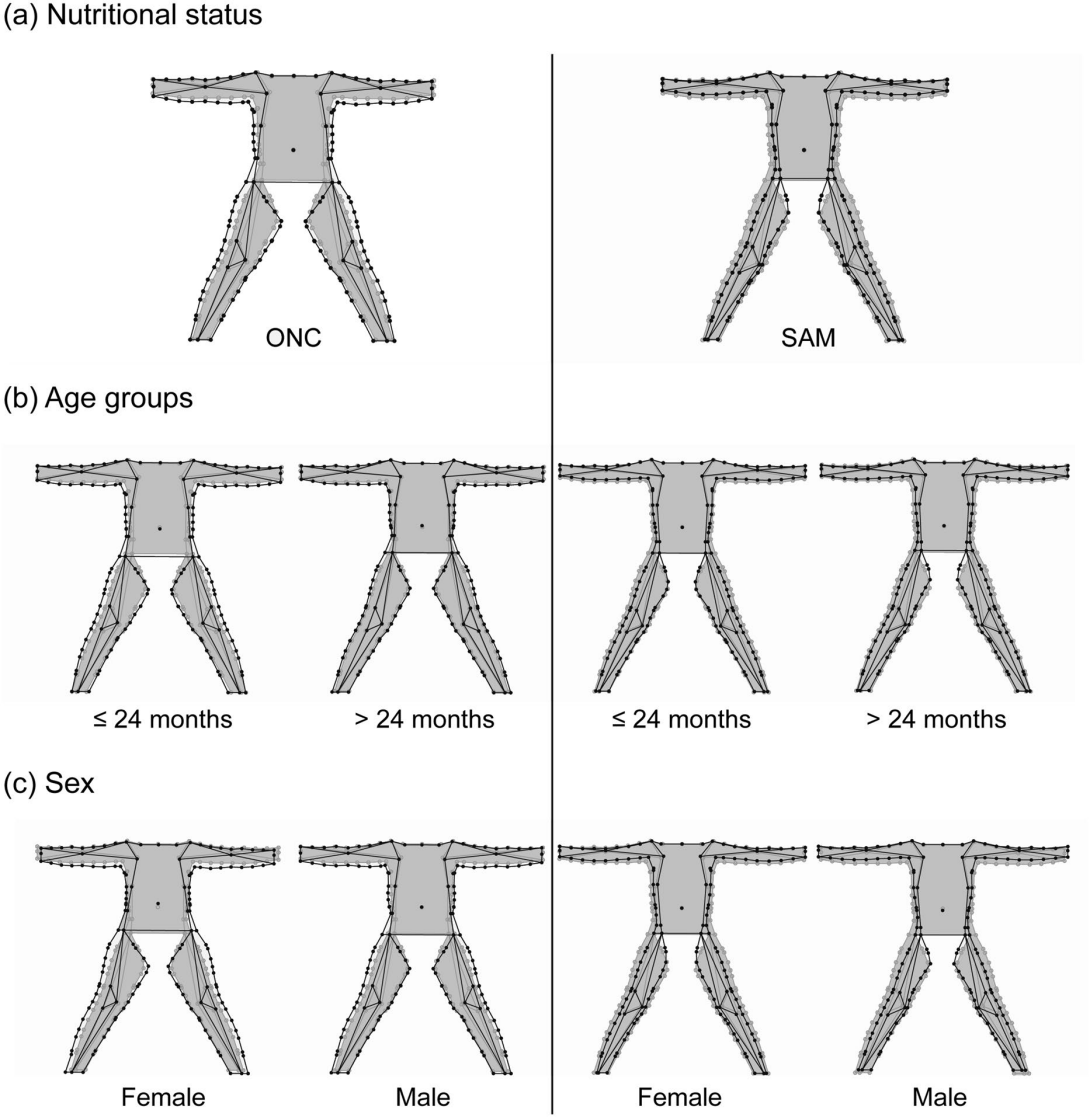
Mean shape variations of body shape in anterior view of infants and children aged 6–59 months before correcting for allometry. Consensus shape (grey) superimposed on the mean shape (black) for each level of the factors: nutritional status (**a**), age groups (**b**) and sex (**c**).

**Figure 3:**
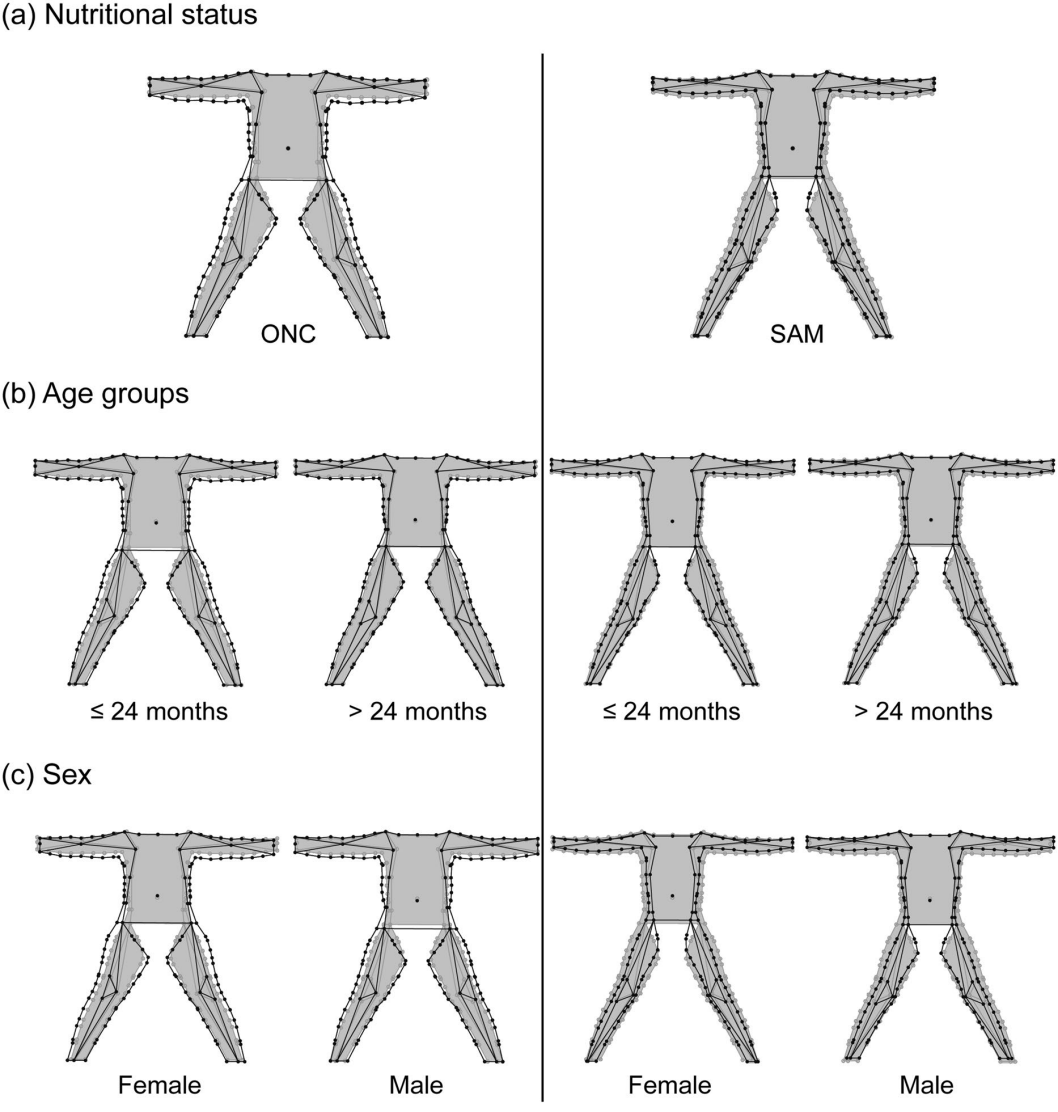
Mean shape variations of body shape in anterior view of infants and children aged 6–59 months after correcting for allometry. Consensus shape (grey) superimposed on the mean shape (black) for each level of the factors: nutritional status (**a**), age groups (**b**) and sex (**c**).

With respect to the effect of wasting on the body shape of participants, mean shapes of ONC and SAM children were compared, considering both age and sex factors to visualize shape changes (Figs. 2, 3). Malnourished children show a general body-thickened shape with respect to ONC children, especially pronounced after 24 months of age. Infants under 24 months show a loss of body mass in all the medial area of the arms both related to the humerus and ulna bones, while children over 24 months exhibit a differential body mass reduction. While tissues related to humerus appear strongly reduced, those disposed in the forearm are less pronounced. When the shape of legs is considered, children under 24 months of age showed a body mass reduction in all the longitudinal distal area as well as in the superomedial half portion of the legs associated to femur bone. On the other hand, older children exhibit a different decrease pattern for the body mass in both distal and medial portions of the inferior limbs.

When body regions were analyzed independently, significant differences (p < 0.01) were found between ONC and SAM participants, both before and after size correction, in the whole sample as well as in both age groups and sexes (Table 3). Legs were the most significantly influenced by size (> 28.4%; p < 0.0001), followed by arms (> 21.1%; p < 0.0001), while the trunk (3.7%; SSp = 0.071; p < 0.0001) remained significantly less influenced by size (Supplementary Table S1). DFA offered correct classification rates over 71.7% and 73.3% before and after correcting for allometric effect respectively when a CV was carried out. The highest accuracy for SAM classification was described in left arm and right leg (both 89.2%) when the whole sample was considered and allometric effect was reduced, resulting in the left arm being the body region which presented higher levels of sensibility (84.3%) and specificity (89.4%). When participants were grouped by age, all body regions showed significant differences between means for ONC and SAM (p < 0.0001) with and without allometric effect influence (Table 3). Under and over 24-month old participants exhibited their best accuracy of correct classification of individuals for the left arm after conducting size correction (88.3% and 95.8%, respectively). In the case of participants aged over 24 months, individuals with SAM were 100% correctly classified and the discriminant function showed the highest sensibility (92.3%) and specificity (100%) ratios among all body regions. The accuracy of DFA was higher in left arm for girls (92.5% correctly classified) and boys (83.3%) after conducting allometric correction, resulting in 93.3% SAM girls and 88.3% SAM boys correctly classified. Classification precision did not improve previous results for any possible combination of sex and age group (Supplementary Table S3).

**Table 3:** Ontogenetic and sexual effect on nutritional status determination in body regions.

Region	Allometric effect	Age group									Sex								
		Factor	Hotelling t^2^ test			DFA + CV Classificaton results (%)					Factor	Hotelling t^2^ test			DFA + CV Classificaton results				
		Age group	n	PrD	*t*^2^	ONC	SAM	Total	Sp	Se	Sex	n	PrD	*t*^2^	ONC	SAM	Total	Sp	Se
Right arm	Before size correction	< 24 months	120	0.028	186.6354*	73.333	70.000	71.667	70.968	72.414	Female	120	0.039	259.25*	76.667	78.333	77.500	77.966	77.049
		> 24 months	120	0.043	523.154*	83.333	90.000	86.667	89.286	84.375	Male	120	0.032	269.709*	80.000	85.000	82.500	84.211	80.952
		Total	240	0.034	369.796*	84.167	83.333	83.750	83.471	84.034	Total	240	0.034	369.796*	84.167	83.333	83.750	83.471	84.034
	After size correction	< 24 months	120	0.044	314.588*	85.000	80.000	82.500	80.952	84.211	Female	120	0.046	521.966*	90.000	86.667	88.333	87.097	89.655
		> 24 months	120	0.045	1059.522*	91.667	96.667	94.167	96.491	92.063	Male	120	0.042	354.698*	83.333	80.000	81.667	80.645	82.759
		Total	240	0.043	695.954*	94.167	87.500	90.833	88.281	93.750	Total	240	0.043	695.954*	94.167	87.500	90.833	88.281	93.750
Left arm	Before size correction	< 24 months	120	0.030	256.642*	78.333	78.333	78.333	78.333	78.333	Female	120	0.041	404.59*	78.333	90.000	84.167	88.679	80.597
		> 24 months	120	0.044	516.957*	85.000	93.333	89.167	92.727	86.154	Male	120	0.032	212.777*	80.000	76.667	78.333	77.419	79.310
		Total	240	0.036	340.468*	79.167	82.500	80.833	81.897	79.839	Total	240	0.036	340.468*	79.167	82.500	80.833	81.897	79.839
	After Size correction	< 24 months	120	0.046	406.152*	93.333	83.333	88.333	84.848	92.593	Female	120	0.048	627.489*	91.667	93.333	92.500	93.220	91.803
		> 24 months	120	0.045	951.096*	91.667	100.000	95.833	100.000	92.308	Male	120	0.042	430.934*	78.333	88.333	83.333	87.037	80.303
		Total	240	0.045	701.924*	91.667	89.167	90.417	89.431	84.252	Total	240	0.045	701.924*	91.667	89.167	90.417	89.431	84.252
Trunk	Before size correction	< 24 months	120	0.070	292.668*	81.667	81.667	81.667	81.667	81.667	Female	120	0.076	338.029*	78.333	83.333	80.833	82.456	79.365
		> 24 months	120	0.073	436.387*	80.000	81.667	80.833	81.356	80.328	Male	120	0.066	254.433*	66.667	80.000	73.333	76.923	70.588
		Total	240	0.071	415.112*	81.667	83.333	82.500	83.051	81.967	Total	240	0.071	415.112*	81.667	83.333	82.500	83.051	81.967
	After size correction	< 24 months	120	0.070	282.604*	70.000	76.667	73.333	75.000	71.875	Female	120	0.077	290.622*	78.333	80.000	79.167	79.661	78.689
		> 24 months	120	0.073	395.383*	78.333	78.333	78.333	78.333	78.333	Male	120	0.065	209.345*	61.667	71.667	66.667	68.519	65.152
		Total	240	0.071	337.819*	79.167	77.500	78.333	77.869	78.814	Total	240	0.071	337.819*	79.167	77.500	78.333	77.869	78.814
Right leg	Before size correction	< 24 months	120	0.047	787.695*	75.000	80.000	77.500	78.947	76.190	Female	120	0.051	1155.900*	81.667	83.333	82.500	83.051	81.967
		> 24 months	120	0.047	1271.527*	73.333	95.000	84.167	93.617	78.082	Male	120	0.044	661.862*	66.667	80.000	73.333	76.923	70.588
		Total	240	0.046	700.175*	80.833	87.500	84.167	86.607	82.031	Total	240	0.046	700.175*	80.833	87.500	84.167	86.607	82.031
	After size correction	< 24 months	120	0.069	760.631*	75.000	80.000	77.500	78.947	76.190	Female	120	0.062	1149.068*	86.667	75.000	80.833	77.612	84.906
		> 24 months	120	0.051	1625.180*	78.333	96.667	87.500	95.918	81.690	Male	120	0.058	637.464*	73.333	70.000	71.667	70.968	72.414
		Total	240	0.059	813.829*	89.167	89.167	89.167	89.167	89.167	Total	240	0.059	813.829*	89.167	89.167	89.167	89.167	89.167
Left leg	Before size correction	< 24 months	120	0.045	577.000*	73.333	78.333	75.833	77.193	74.603	Female	120	0.049	1161.578*	86.667	81.667	84.167	82.540	85.965
		> 24 months	120	0.047	1396.813*	86.667	85.000	85.833	85.246	86.441	Male	120	0.043	585.760*	63.333	73.333	68.333	70.370	66.667
		Total	240	0.045	656.507*	80.000	80.833	80.417	80.672	80.165	Total	240	0.045	656.507*	80.000	80.833	80.417	80.672	80.165
	After size correction	< 24 months	120	0.067	662.661*	75.000	73.333	74.167	73.770	74.576	Female	120	0.060	999.605*	88.333	80.000	84.167	81.538	87.273
		> 24 months	120	0.051	1664.169*	91.667	91.667	91.667	91.667	91.667	Male	120	0.057	506.725*	70.000	68.333	69.167	68.852	71.186
		Total	240	0.058	687.322*	87.500	81.667	83.333	80.769	86.364	Total	240	0.058	687.322*	87.500	81.667	83.333	80.769	86.364

Discriminant Function Analysis (DFA) cross-validated (CV) results for nutritional status determination in body regions of children considering age groups and sex independent effect.

Procrustes distance (PrD), Hotelling t^2^ statistic (t^2^). (*) Significance level: p < 0.001. Optimal nutritional condition (ONC), severe acute malnutrition (SAM). Specificity (Sp) and sensibility (Se) estimated for SAM.

## Discussion

This article presents a pioneering application of GM techniques to study nutritional status by means of 2D images of children’s body shape registered in anterior view. The methodology here deployed was first applied to ONC Senegalese and Spanish children aged 6–59 months, to describe ontogenetic, sexual and populational effects on morphology^17^. Taking such effects into account, the present work explores nutritional status effects on shape and size variations of Senegalese children. Here we have analyzed both SAM and ONC shape patterns, taking into account ontogenetical and sexual factors.

WHO currently supports community-based care for children with uncomplicated SAM, which has enhanced health facilities to better invest their resources in handling SAM children with complications^7^. Moreover, community management of SAM is increasing worldwide, boosting the number of affected children who are being reached, and making this intervention more cost-effective^5^. However, the generalized use of the current anthropometric indicators of WHZ and MUAC at community level is prone to several limitations. On one hand, there seems to be a disagreement about whether both indicators are appropriate for their implementation in the community. MUAC seems more suitable to be used at community level due to its simplicity^8,9^ and has actually become the primary identification tool for SAM^23^. Furthermore, since WHZ assessment is more time consuming^8^, some LMIC national policies suggest using just MUAC and oedema for admission and discharge in cases of inadequate availability of resources or restricted to inpatient care^2,3,23^. MUAC and WHZ, however, do not overlap uniformly when identifying SAM, meaning that they detect different sets of infants and children^11,24^. Thus, the use of only one of such criteria implies that a significant number of infants and children in need for treatment could be left without treatment and many researchers strongly recommend that both MUAC and WHZ be used^11^. While there is still a need to unify the way of assessing SAM, the fact that each indicator targets different individuals requires deeper research towards its biological and physiological implications.

On the other hand, the current lack of resources in many LMIC health facilities leads to shifting the responsibility of screening for nutritional status onto community health workers, para-professionals who participate in health promotion and disease prevention including basic treatment and collecting community health information^25^. The fact that the current WHZ and MUAC indicators used to assess SAM are based on anthropometric measurements leads to high intra- and inter-observer errors. Anthropometry is a systematized method which requires highly trained people to use standardized instruments and techniques to locate anthropometric points and measure between them as accurately as possible^10^. Moreover, when several observers collect anthropometric data in the same set of subjects, interobserver error needs to be estimated and minimized^10^. However, assessing interobserver error may be very challenging at community level since people registering the measurements may be far from each other and not even in professional contact with each other. Furthermore, appropriate materials used to register the measurements are not always available in such contexts and health facilities have to improvise them, not allowing to guarantee the accuracy of the measurements when replicating WHO methods^6^. Thus, anthropometric data assessment of SAM may include intra- and interobserver errors which bias the obtained results, even if the methods themselves are useful. Taking into consideration that SAM is an extreme situation which requires immediate action, the use of such methodology under current conditions of deployment is not appropriate at community level.

The methods proposed in this article present an objective way of assessing SAM by means of shape variables. This innovative methodology provides several advantages over classical anthropometric methods. First, data collection is very rapid. Since many infants and young children are unlikely to tolerate manual measurements and remain calm and still during the data collection process, this feature is especially valuable in this age group^15^. Second, a wide range of shape variables can be extracted, allowing complex multivariate analysis of data, and electronically stored for further research and assessment. Third, it allows shape change visualization, increasing knowledge available on infant and child body composition and tissue distribution regarding SAM. Fourth, GM methods have demonstrated to be highly replicable due to the reduced intra- and interobserver errors^26^. Fifth, the use of landmark-based variables permits the construction of models which can be very informative to evaluate shape changes accumulated as a consequence of the effect of single or multiple factors^18,26^. Sixth, such study could be complemented by analyzing morphometric variations during different periods of time by superposing configurations of landmarks of the same subjects over time.

Therefore, several studies have used image-based technologies to study adults, but seldom teenagers and infrequently infants and children^14,16,17,20–22,27^. Most research has shown substantial shape variability in association with age, gender and ethnicity^17,21^. At present, this work demonstrates that GM techniques are a suitable collection of tools to study nutritional status morphological variations during childhood, and also provides the first evidence on SAM shape patterns of infants and children under 5 years old considering age and sex effects as recommended by previous works^6^.

Body size and its proportions change in an expectable and apparent way during growth, closely related to functional or motor development, and often in alternating rapid and slow growth rates^4,27–29^. Growth and developmental rates vary from one part of the body to another and among different tissues. Even a single tissue, such as adipose tissue, does not grow in a uniform manner and this creates patterns of body fat distribution. Body fat distribution has been associated with nutritional status, socioeconomic status, sex, age, and ethnicity^30^. Our results describe such changes in terms of shape variations from infancy to early childhood. These fluctuations, previously described in the literature as changes in anthropometrical parameters, are here visualized and described in comparison to a consensus shape extracted from the data set. Thus, our results confirm that trunk and limb dimensions and shape show an opposite distribution before and after 24 months of age, primarily due to starting to walk^17^. In addition, ontogenetic patterns seem similar among SAM and ONC children, since both samples show the same morphological variations related to the infant and child growth stages. In both samples, our results confirm previous works on body composition and metric data^4,12,28,30^ or landmark-based analysis^17^. Infants under 24 months old have longer arms with respect to their legs and their trunk is wider at the shoulders than at the chest^12^. After 24 months of age, leg length and breadth becomes progressively larger, legs become longer than arms and chest dimensions remain similar confirming previous research which attributed such morphology to the lowered position of liver and kidneys in the abdominal cavity^31^.

Ontogenetic effect seems to have a great influence in shape components, as both morpho-spaces constructed, even with adjustment for allometric effects, depict significant differences for such a factor. Moreover, allometric changes during growth are evident in our sample, since once the size effect has been reduced, no significant changes are described between different age groups and non-allometric significant shape changes remain uncertain along PCs. These results, on the one hand, confirm that the study of ontogenetic shape variation requires considering the effect of size^17^ and, on the other hand, the results indicate that by mitigating age effects with the correction for allometry, other factors whose effect may possibly be masked by the influence of ontogeny may become more evident. In the present analysis, an example of this second statement is nutritional status. Although there are significant differences among SAM and ONC infants and children in body shape and size in the presence of the allometric factor, after size adjustment the nutritional status effect is increased.

Our work localizes those regions of the body where the nutritional effect is more evident and points out differences in morphology between SAM and ONC children that seem to be related to variations in the rate of fat and lean deposits. These data may be valuable for understanding the early tissue accretion patterns that characterize wasted children in contrast to those who do not present such pathology. They may be also useful for proposing the study of other body regions, in addition to MUAC, when addressing acute malnutrition and thus enhancing the knowledge on wasting provided by current indicators used.

Sexual dimorphism has been confirmed in infants and children under five by means of their body shape^4,15,27,30,32^. Considering overall relative size of the body, the present study reveals no significant differences between girls and boys (Table 1). Since ontogenetic effects are known to influence relative size changes of the body of children, before allometric correction, it is very possible that such effects could be masking the expression of other factors such as sexual dimorphism. This pattern remains the same between ONC and SAM children, demonstrating that the age effect represents a similar magnitude in both groups regardless nutritional status. Infants and children under and over 24 months of age do not exhibit sexual dimorphism for the size component either, meaning that sex effect on CS for the whole body may be minor in the sample analyzed. These results coincide with findings made by other authors who report minor differences during infancy and childhood, but more important sex differences with the onset of puberty^4,15,30,32^.

With respect to body shape, we report significant sex differences both considering and minimizing allometric effect in the global sample, both age groups, SAM children, and only in ONC participants after the adjustment for the size effect. From these results there are two main considerations to be highlighted. The first is the fact that sexual dimorphism of children’s body shape is highly influenced by the size effect. This assumption has already been observed when analyzing other structures of the body with GM methods such as the facial soft tissue^33^ and the skull^34^ in children. These studies also report non-allometric shape differences related to a differential sexual expression. Our results have provided a concrete non-allometric sexual dimorphism pattern in the body of children. The second is that SAM participants show a significant allometric sexually dimorphic pattern while ONC participants do not show it, demonstrating the slight influence of sex in the morphometric variability of the body when size effect is considered. Thus, changes in infant and young child body shape related to nutritional deprivation may be more important than sex differences.

Sexual morphometrical variation in the body of the children seem to be highly related to allometry. However, it is also demonstrated in the present study that some morphological changes in boys related to ontogeny are exclusively based in non-allometric shape components. With this respect, further research on the relationship between body composition or anthropometric measurements with morphological variations described in this work could help understanding the contribution of the different tissues conforming the overall body shape to its morphological patterns in girls and boys during growth.

Our work shows that age and sex are two factors that need to be taken into consideration when classifying nutritional status of infants and children. Without consideration of age and sex the classification of individuals is no better than chance. If ontogenetic morphometrical variations are considered, it is evident that the morphometric pattern of SAM children under 24 months and ONC children over 24 months may distort the discriminant function construction resulting in a misclassification. Similarly, female and male SAM and ONC patterns may somehow intersect to a certain degree. These statements are confirmed by the higher classification accuracies obtained when the sample is subdivided by age groups (80–92.5%), sexes (75–80%), or when both factors are combined to subdivide the sample in four groups (70–98.3%). Furthermore, the allometric effect shows a great but differential influence in the ability of the discriminant functions constructed to correctly classify SAM and ONC children. For example, when the sample is subdivided by age groups, correct classification rates provided by shape variables after allometric correction increase, meaning that once the ontogenetic effect is minimized in different stages of growth and development, shape variability due to other factors such as nutritional status may emerge.

Our results confirm that children’s body shape changes with the adoption of an erect posture. This may explain why in infants and children over 24 months the accuracy of the classification algorithm is better (92.5%) than for younger infants (89.7%). Infants and children over 24 months presumably have acquired an upright posture and move bipedally^28,29^. The group of infants under 24 months is composed by babies at various levels of crawling and others who have already assumed an erect posture. It is very possible that the morphologic pattern associated to each of these locomotion modalities may be overlapping with the expression of the different nutritional statuses, decreasing the efficiency of the classifier in this group.

If different anatomical regions are considered independently, nutritional status can also be predicted accurately. The upper limbs offer the best results (> 90%) once the allometric effect has been minimized. For infants and children over 24 months, the correct classification of SAM, after size correction, reaches 100% for the left arm. In the study of the whole-body structure, different effects such as those exerted by ontogenetic, sexual or allometric factors are expressed in a differential manner in diverse body regions. Such effects could be producing an accumulative effect on the morphometrical changes among infants and children that is not due to nutritional status variations. Thus, when body regions are analyzed independently, the variability not associated to nutritional status may be reduced, allowing a better classification of the individuals. It is therefore essential to complement the results of the present study with further research into the morphological integration of the different structures in the whole body and the effects that age, sex and size may have in the covariation among these structures.

The model described in this study is limited by the characteristics of the sample, chosen as a convenience sample due to its distribution by age, sex and nutritional status spectrum. However, we have accomplished our objective of describing morphometrical patterns of SAM in children under five. Certainly, the development of large-scale studies, including more nutritional status typologies (from obesity to undernutrition) will be essential to build a database for the interpretation, analysis and classification of different morphological trends. Moreover, the relation between the current anthropometric-based criteria used to assess acute malnutrition (WHZ and MUAC) and shape variables describing the morphology of children under 5 years old need to be analyzed in future studies with individuals showing different degrees of acute malnutrition. In addition, since body shape differences between ethnic groups are already established at young ages^4,21^, this methodology will need to be applied to children living in different worldwide contexts to analyze the effect of population-specific factors in the phenotypic expression of different nutritional statuses.

In conclusion, the final goal of our ongoing project is to develop a smartphone application to be used by community health workers which can estimate SAM by means of GM techniques. The present article provides proof of concept of the pertinence of such methods to assess nutritional status in children under 5 years old. Moreover, this study provides evidence on the differential growth and development patterns of body shape of infants and children when nutritional status is considered, highlighting the influence of age and sex as main populational factors, together with allometric effect, as intrinsic factor related to the development of effective GM methods.

## Materials and methods

### Experimental design

#### Sample

A sample of 240 Senegalese infants and children (120 girls and 120 boys) aged 6–59 months was studied from November 2016 to May 2017 framed in the research project “*Réinventer la gestion communautaire de la malnutrition aiguë: Diagnostic par image de la malnutrition aiguë sévère.* (CMAM 2.0)”. This project aims to develop a pioneer smartphone tool for identifying malnutrition based on child shape by means of a picture of a body region, applying a methodology previously designed^17^. These methods were first validated in a ONC sample of Senegalese and Spanish children aged 6–59 months^17^ and applied in the present study to better understand morphological body patterns defined by nutritional status. The sample was collected in schools, nurseries and communities in Matam, one of the regions in the country with higher SAM prevalence^35^. Selection criteria consisted in: (a) having Senegalese origin, at least three generations including the child under study (important for future scaling up studies regarding populational effect on nutritional status identified by GM techniques); (b) age between 6 and 59 months; (c) absence of health complications (no clinically diagnosed condition other than SAM), marked skeletal deformities or musculoskeletal injuries or disabilities which may concern body shape; (d) absence of nutritional bilateral oedema, since it is considered a health complication which require immediate hospitalization; and (e) ONC with WHZ between − 0,5 SD and 0.5 SD or MUAC between 135 and 165 mm, or SAM condition, determined according to the World Health Organization (WHO) growth standards^7^. Caregivers of all children participating in the study gave written, informed consent and the study was approved by National Ethical Committee for Health Research (CNERS), Ministry of Health and Social Action (Dakar, Senegal). Privacy data protection was ensured according to the Senegalese and European data protection laws.

For addressing age effect, two age groups were designed according to WHO categorization of children under 5 years old based in the way of registering length or height: (1) infants under 24 months (n = 131), recumbent and (2) children over ( ≥) 24 months (n = 171), standing^6^. To ensure a homologous representation of all ages in those two age groups, the experimental design included a clustering of infants and children between 6 and 59 months into five age ranges as follows: 6–12 months, 13–24 months, 25–36 months, 37–48 months and 49–59 months. Thus, the sample was equally distributed for age (under and over 24 months) and sex in groups of 60 children each (Supplementary Table S4). Regarding nutritional status, each group was subdivided in ONC or SAM condition, accounting for 30 children each.

### Anthropometric measurements

To identify the nutritional status of the participants, anthropometric measurements of weight, height, and MUAC were registered. To ensure the accuracy of the measurements and the ultimate image capture if they accomplished the enrolling criteria, participants were undressed to their underwear or tight swimming costume bottom. Weight was measured with a portable electronic scale Soehnle, calibrated to 0.1 kg. A portable infantometer (range 10–100 cm to a 0.1 cm precision level) was used to measure length in children under 87 cm or 24 months of age (closed interval for both factors), while height was measured in children over 87 cm or 24 months of age by an anthropometer GPM (range 10–230 cm, with 0.1 cm precision level). Finally, MUAC was measured through a self-retracting, 0.7 cm-wide, flat metal tape with a blank lead-in strip (with a precision level of 1 mm). Before starting each data collection session, calibration of scales and substitution of damaged measuring tapes was carried out.

### Nutritional status estimation

Just after registering the measurements, the R package “Anthro” provided by WHO^36^ was used to calculate WHZ, as well as other nutritional status indicators (descriptive analysis provided in Supplementary Table S5). Generated data were evaluated just after recording the measurements, and WHZ and MUAC nutritional indicators were estimated, considering SAM those children with WHZ < − 3 SD or MUAC < 125 mm. While ONC is considered between percentiles 15 and 85 for WHZ (between − 1 and 1 SD) and MUAC ≥ 125, only children between the 30th and 70th percentiles (− 0.5 and 0.5 SD) for at least one of the indicators were enrolled with ONC in the present study. The aim was to reduce as much as possible the morphological heterogeneity associated with nutritional status. All caretakers of children participating in the study received a nutritional status assessment regarding to their children by means of a written or oral report. Every child included in the study was given an identification code to ensure health information (registered in a database) and eventual image privacy.

### Shape data acquisition

Following the methodology previously designed, a set of 34 points were anatomically identified by palpation and marked with dermatologically tested pens on the children's anterior body in order to properly determine the subsequent landmarks position^17^. Children were then placed in a determinate position (laying down forwards on a mat with a template to place them with arms perpendicular to the body and legs opened 30°) to register images of their body in anterior view using a digital reflex camera Nikon D3300 and a Manfrotto tripod. There were three people holding the child, one the feet, another one the hands and a third one the head. Positioning followed the previous methods designed for this purpose and always included the placing of a size scale and a code in the scenario, appropriate for later data management (for further details please check reference 17). A sequence of at least 15 pictures were taken of each participant, under conditions that offered them the least stress due to the fact of having to remain immobilised. It is important remark that the methodology developed considered the fact that subjects under study were infants and children, who easily get tired of remaining in the same position and often refuse to collaborate, especially when they are too young and unable to understand the purpose of the tasks they are required to cooperate with. This fact is important since it is often a limitation in studies with infants and children, that generally require minimum developmental maturity to be performed^15,16^. In the present case, our method allows the study infants and children from 6 months old, and it could be applicable to eventually younger, depending on the research requirements. As detailed in our previous methodological work^17^, recording anthropometric measurements, marking points on the body and taking pictures of children can be a difficult experience for them. The procedures needed to implement the methods here described were adapted when working with communities to provide a comfortable environment for all children, families and researchers, involving them at different levels during all the data collection process to create a calmed and confident atmosphere. We waited patiently for children to be relaxed to interact with them and we invented games to get children familiar with the examiners. It is important to mention that families' participation in such kind of studies is voluntary and that, in certain contexts, access to health assistance is reduced and our intervention may result crucial for providing knowledge to families.

A picture selection was performed from each individual's registered picture sequence and selected images were processed with Gimp 2.8 to remove shadow and orientation effects as well as to identify points marked on the body of the children and perform the geometric calculations required to replicate the template^17^. Landmark and semi-landmark configurations registration were then accomplished using tpsDig2 software^37^.

### Statistical analyses

GM methods were applied to visualize major shape changes as landmark-based shape deformations, using thin-plate spline^18^, and to perform multivariate analyses on landmark coordinates, to analyze the effect of relative size, sex and age (as a continuous variable and grouped by under and over 24 months) on the children's anterior view whole body shape depending on their nutritional status, using MorphoJ^38^ and R software. Raw configurations of landmarks were normalized using Generalized Procrustes Analysis (GPA) to remove size as a factor, although size—CS-related shape differences may remain^18,26^. Thus, a multivariate regression of remaining shape variables (Procrustes coordinates) on the logarithm of CS (log CS) was performed to assess allometry effect. A 1000 random repetitions permutation test was performed within the multivariate regression to assess its statistical value and the effect of allometry in the data was finally corrected employing multivariate regression residuals as shape variables independent from CS effect^39^.

A Procrustes ANOVA (p < 0.05) was performed, before and after the allometric correction, to explore the effect of age (continuous and grouped), sex and nutritional status (grouped in SAM and ONS) in both shape and size^40^. In addition, a principal components analysis of the shape variables, before and after the size correction, was carried out in order to obtain a set of basis vectors describing major trends in shape variation^18,26^. PC scores for the first two PCs were depicted in a scatter plot using confidence ellipses (confidence of 90%) to group individuals by means of age groups, sex and nutritional status. Shape differences were visualized in terms of differences in the deformation grids depicting the objects.

A DFA was performed on all whole body and body regions datasets (with and without allometric correction) in order to classify individuals by means of their nutritional status. A cross validation leave-one-out testing method was applied to address the accuracy of the DFA and to approach the capability of the proposed landmarks templates to estimate SAM in other populations. Specificity and sensitivity for SAM classification were also estimated. A complementary t^2^ Hotelling test was carried out to estimate differences between means, including a permutation test (10,000 runs) in the whole-body datasets due to the high number of variables (144) of such template.

## Supplementary Information

Supplementary Information.

## Abbreviations

CNERS: National Ethical Committee for Health Research
CS: Centroid size
CV: Cross validation
DFA: Discriminant function analysis
GM: Geometric morphometrics
GPA: Generalized procrustes analysis
LMIC: Low- and middle-income countries
Log CS: Logarithm of centroid size
MUAC: Mid-upper-arm circumference
ONC: Optimal nutrition condition
PC: Principal component
PCA: Principal component analysis
SAM: Severe acute malnutrition
SD: Standard deviations
WHO: World Health Organization
WHZ: Weight-for-height/length z-score
