# Supplementary material for: Severe acute malnutrition morphological patterns in children under five
Source: Sci Rep. 2021 Feb 19;11:4237. doi: 10.1038/s41598-021-82727-x (PMC7895927; doi:10.1038/s41598-021-82727-x)
Supplement: Supplementary file 1 — Supplementary Information. [file 41598_2021_82727_MOESM1_ESM.pdf]

Supplementary Materials.

Severe acute malnutrition morphological patterns in children under five

Laura Medialdea, Barry Bogin, Mbeugue Thiam, Antonio Vargas, María D. Marrodán, Nicole I. Dossou

Materials and Methods

Table S1. Sample composition. Infants and children aged 6-59 months with an optimal nutritional condition (ONC) or severe acute malnutrition (SAM) analyzed in this work.

Age (months)		ONC			SAM			Total
		F	M	Total	F	M	Total	
< 24	6-12	10	9	19	11	12	23	42
	13-24	20	21	41	19	18	37	78
	Total	30	30	60	30	30	60	120
> 24	25-36	8	5	13	5	3	5	18
	37-48	13	10	23	10	10	15	38
	49-59	9	15	24	15	17	40	64
	Total	30	30	60	30	30	60	120
Total		60	60	120	60	60	120	240

Table S2. Anthropometrical measurements descriptive analysis. Descriptive analysis of anthropometrical measurements recorded in infants and children aged 6-59 months with an optimal nutritional condition (ONC) or severe acute malnutrition (SAM).

	ONC					
	< 24 months			> 24 months		
Variable	Girls	Boys	Total	Girls	Boys	Total
Age (months)	22.0 ± 13.6	23.0 ± 13.0	22.5^ ± 13.2	34.6 ± 16.7	37.5 ± 13.2	36.0^ ± 15.0
Weight (Kg)	8.9 ± 1.2	9.2 ± 1.3	9.1*^ ± 1.3	**13.7 ± 2.0**	**15.2 ± 2.1**	14.4*^ ± 2.1
Height (cm)	75.6 ± 5.6	76.3 ± 6.2	76.0*^ ± 5.9	**96.7 ± 7.5**	**101.1 ± 7.3**	99.0^ ± 7.7
MUAC (cm)	14.5 ± 0.8	14.5 ± 0.5	14.5*^ ± 0.7	15.8 ± 0.7	16.2 ± 0.9	16.0*^ ± 0.8
WHZ	-0.54 ± 0.89	-0.56 ± 0.67	-0.55* ± 0.78	-0.57 ± 0.55	-0.47 ± 0.68	-0.52* ± 0.61
WAZ	-0.35 ± 0.89	-0.79 ± 0.81	-0.56* ± 0.87	-0.67 ± 0.79	-0.44 ± 0.83	-0.55* ± 0.82
HAZ	0.13 ± 1.40	-0.40 ± 1.23	-0.13* ± 1.33	-0.44 ± 1.27	-0.17 ± 1.37	-0.31* ± 1.32
MUACAZ	**0.13 ± 0.70**	**-0.20 ± 0.50**	-0.03* ± 0.63	0.00 ± 0.44	0.08 ± 0.53	-0.04* ± 0.48
	SAM					
	< 24 months			> 24 months		
Variable	Girls	Boys	Total	Girls	Boys	Total
Age (months)	20.2 ± 14.3	20.9 ± 12.2	20.6^ ± 13.2	**44.4 ± 15.6**	**35.2 ± 16.7**	39.8^ ± 16.7
Weight (Kg)	5.7 ± 1.0	6.0 ± 1.3	5.8*^ ± 1.1	10.9 ± 1.2	11.0 ± 1.2	10.9*^ ± 1.2
Height (cm)	68.5 ± 5.2	68.3 ± 6.3	68.4*^ ± 5.9	97.6 ± 6.6	96.5 ± 6.4	97.1^ ± 6.4
MUAC (cm)	**10.7 ± 0.7**	**11.0 ± 1.0**	10.8*^ ± 0.8	11.6 ± 0.5	11.8 ± 0.3	12.3*^ ± 0.4
WHZ	-3.69 ± 0.67	-3.57 ± 0.56	-3.63* ± 0.62	-3.96 ± 0.46	-3.47 ± 0.40	-3.43* ± 0.43
WAZ	-3.93 ± 1.19	-4.23 ± 1.18	-4.08*^ ± 1.18	-3.06 ± 0.65	-3.02 ± 0.72	-3.04*^ ± 0.68
HAZ	**-2.32 ± 1.70**	**-3.16 ± 1.85**	-2.74*^ ± 1.81	-1.34 ± 1.25	-1.34 ± 1.12	-1.34*^ ± 1.17
MUACAZ	-3.48 ±0.47	-3.69 ± 0.67	-3.58* ± 0.58	-3.73 ± 0.43	-3.59 ± 0.27	-3.66* ± 0.35

Significance level: (*) $p < 0.05$ for comparison between ONC and SAM children in each age group; (^) $p < 0.05$ for comparison between children under and over 24 months in each nutritional status group; bold for comparison between sexes in the same age and nutritional status group.

Results

Table S3. Allometric effect on shape variability of the whole body and body regions. Regression analysis of shape on size to quantify allometric effect among different body regions as well as on the whole body.

Regression analysis					
Configuration	SSp	SSr	SS	Pvar (%)	p-value
Whole body	0.243	0.453	0.696	34.920	<0.0001*
Left arm	0.211	0.718	0.929	22.733	<0.0001*
Right arm	0.192	0.716	0.909	21.174	<0.0001*
Trunk	0.071	1.849	1.919	3.674	<0.0001*
Left leg	0.400	0.953	1.353	29.575	<0.0001*
Right leg	0.391	0.987	1.378	28.402	<0.0001*

Predicted (SSp), residual (SSr) and total (SS) Procrustes sums of squares; proportion of variation for which the regression accounts with respect to the total variation (Pvar). (*) Significance level after 10.000 rounds permutation test against null hypothesis of independence.

Table S4. Effect of the variables under study on children's whole-body shape and size. Procrustes ANOVA test for shape (Procrustes coordinates) and size (log centroid size) by means of age groups, sex, and nutritional status for the whole-body template subdividing the sample in different subsets according to factor's levels.

			Shape					Size				
Sample	Procrustes ANOVA	Effect	SS	MS	df	F	p-value	SS	MS	df	F	p-value
ONC	Before Size correction	Age Group	9.079E-02	3.197E-04	284	48.53*	<0.0001	1.753E+07	1.753E+07	1	264.15*	<0.0001
		Sex	1.685E-03	5.932E-06	284	0.90	0.8836	6.253E+04	6.253E+04	1	0.94	0.3337
	After Size correction	Age group	5.757E-03	2.027E-05	284	3.47*	<0.0001	9.000E-06	9.000E-06	1	26.90*	<0.0001
		Sex	2.201E-03	7.751E-06	284	1.33*	0.0002	<0.001E-4	<0.001E-4	1	0.19	0.6676
SAM	Before Size correction	Age group	1.296E-01	4.565E-04	284	73.44*	<0.0001	3.043E+07	3.043E+07	1	422.98*	<0.0001
		Sex	4.596E-03	1.618E-05	284	2.60*	<0.0001	1.581E+04	1.581E+04	1	0.22	0.6401
	After Size correction	Age group	1.318E-03	4.639E-06	284	0.79	0.9966	8.000E-06	8.000E-06	1	19.07*	<0.0001
		Sex	4.132E-03	1.455E-05	284	2.47*	<0.0001	<0.001E-4	<0.001E-4	1	0.01	0.9206
< 24 months	Before Size correction	Sex	3.110E-03	1.095E-05	284	1.35*	<0.0001	2.735E+03	2.735E+03	1	0.04	0.8377
		Nutritional status	2.910E-02	1.025E-04	284	12.67*	<0.0001	2.770E+06	2.770E+06	1	42.66*	<0.0001
	After Size correction	Sex	3.372E-03	1.187E-05	284	1.57*	<0.0001	<0.001E-4	<0.001E-4	1	0.02	0.0882
		Nutritional status	3.051E-02	1.074E-04	284	14.18*	<0.0001	<0.001E-4	<0.001E-4	1	0.00	0.9480
> 24 months	Before Size correction	Sex	2.224E-03	7.831E-06	284	1.64*	<0.0001	5.188E+03	5.188E+03	1	0.07	0.7916
		Nutritional status	1.858E-02	6.541E-05	284	13.72*	<0.0001	1.123E+05	1.123E+05	1	1.52	0.2204
	After Size correction	Sex	2.496E-03	8.787E-06	284	2.10*	<0.0001	<0.001E-4	<0.001E-4	1	1.67	0.1982
		Nutritional status	1.918E-02	6.754E-05	284	16.18*	<0.0001	<0.001E-4	<0.001E-4	1	0.01	0.9358
Female	Before Size correction	Age group	1.029E-01	3.624E-04	284	56.10*	<0.0001	2.344E+07	2.344E+07	1	301.47*	<0.0001
		Nutritional status	2.232E-02	7.859E-05	284	12.17*	<0.0001	6.590E+05	6.590E+05	1	8.48*	0.0043
	After Size correction	Age group	1.732E-03	6.097E-06	284	1.02	0.3931	7.000E-06	7.000E-06	1	27.47*	<0.0001
		Nutritional status	2.586E-02	9.107E-05	284	15.25*	<0.0001	0.000E+00	0.000E+00	1	0.04	0.839
Male	Before Size correction	Age group	1.144E-01	4.029E-04	284	62.57*	<0.0001	2.363E+07	2.363E+07	1	347.08*	<0.0001
		Nutritional status	2.315E-02	8.153E-05	284	12.66*	<0.0001	1.410E+06	1.410E+06	1	20.71*	<0.0001
	After Size correction	Age group	3.437E-03	1.210E-05	284	2.08*	<0.0001	1.000E-05	1.000E-05	1	20.23*	<0.0001
		Nutritional status	2.238E-02	7.882E-05	284	13.54*	<0.0001	0.000E+00	0.000E+00	1	0.04	0.9139

Procrustes sums of squares (SS), Procrustes mean squares (MS), degrees of freedom (df). Goodall's F statistic (F). (*) Significance level: $p < 0.01$.

Table S5. Ontogenetic and sexual combined effect on nutritional status determination in body regions. Discriminant Function Analysis (DFA) cross-validated (CV) results for nutritional status determination in body regions of children considering age groups and sex combined effect.

				Hotelling t^2 test				DFA + CV Classificaton results (%)				
Region	Allometric effect	Sex	Age group	n	PrD	t^2	p-value	ONC	SAM	Total	Sp	Se
Right arm	Before Size correction	Female	< 24 months	30	0.03	214.825	0.0933	60.00	70.00	65.00	66.67	36.36
			> 24 months	30	0.05	866.583**	<0.0001	80.00	90.00	85.00	88.89	81.82
		Male	< 24 months	30	0.03	293.023*	0.0216	60.00	73.33	66.67	69.23	64.71
			> 24 months	30	0.04	443.0722*	0.0019	66.67	73.33	70.00	71.43	68.75
	After Size correction	Female	< 24 months	30	0.046	311.156*	0.0156	60.00	76.67	68.33	72.00	65.71
			> 24 months	30	0.048	1320.604**	<0.0001	76.67	93.33	85.00	92.00	80.00
		Male	< 24 months	30	0.044	397.645**	0.0037	70.00	76.67	73.33	75.00	71.88
			> 24 months	30	0.042	896.332**	<0.0001	80.00	90.00	85.00	88.89	81.82
Left arm	Before Size correction	Female	< 24 months	30	0.032	621.807**	0.0002	73.33	86.67	80.00	84.62	76.47
			> 24 months	30	0.047	509.039**	0.0007	70.00	80.00	75.00	77.78	72.73
		Male	< 24 months	30	0.032	320.798*	0.0132	76.67	66.67	71.67	60.61	74.07
			> 24 months	30	0.035	584.315**	0.0003	70.00	83.33	76.67	80.77	73.53
	After Size correction	Female	< 24 months	30	0.048	812.832**	<0.0001	73.33	86.67	80.00	84.62	76.47
			> 24 months	30	0.050	758.528**	<0.0001	76.67	96.67	86.67	95.83	80.56
		Male	< 24 months	30	0.045	619.731**	0.0002	86.67	86.67	86.67	86.67	86.67
			> 24 months	30	0.041	1031.619**	<0.0001	83.33	90.00	86.67	89.29	84.38
Trunk	Before Size correction	Female	< 24 months	30	0.073	545.367	0.0838	56.67	63.33	60.00	60.71	59.38
			> 24 months	30	0.082	1217.382**	0.0037	70.00	76.67	73.33	75.00	71.88
		Male	< 24 months	30	0.069	370.950	0.2518	53.33	50.00	51.67	51.61	51.72
			> 24 months	30	0.065	690.532*	0.0371	60.00	63.33	61.67	62.07	59.38
	After Size correction	Female	< 24 months	30	0.071	361.236	0.2685	53.33	60.00	56.67	57.14	56.25
			> 24 months	30	0.082	924.622*	0.0120	53.33	76.67	65.00	69.57	62.16
		Male	< 24 months	30	0.070	781.277*	0.0234	60.00	60.00	60.00	60.00	60.00
			> 24 months	30	0.065	521.662	0.0967	56.67	63.33	60.00	60.71	59.38
Right leg	Before Size correction	Female	< 24 months	30	0.047	1300.824	0.8868	73.33	76.67	75.00	75.86	74.19
			> 24 months	30	0.057	2786.687	0.7236	66.67	73.33	140.00	71.43	68.75
		Male	< 24 months	30	0.050	1362.606	0.8784	63.33	60.00	61.67	61.29	62.07
			> 24 months	30	0.041	4405.352	0.6142	70.00	90.00	80.00	87.50	75.00
	After Size correction	Female	< 24 months	30	0.068	1635.270	0.8431	76.67	80.00	78.33	79.31	77.42
			> 24 months	30	0.057	3545.257	0.6659	70.00	63.33	66.67	65.63	67.86
		Male	< 24 months	30	0.070	1495.142	0.8610	56.67	70.00	63.33	60.71	61.76
			> 24 months	30	0.047	4726.122	0.5977	66.67	86.67	76.67	83.33	72.22
Left leg	Before Size correction	Female	< 24 months	30	0.043	1196.324	0.9011	66.67	60.00	63.33	62.50	64.29
			> 24 months	30	0.058	5434.052	0.5654	86.67	96.67	90.00	96.30	87.88
		Male	< 24 months	30	0.048	578.991	0.9809	56.67	63.33	60.00	60.71	59.38
			> 24 months	30	0.041	2449.655	0.7540	66.67	70.00	68.33	68.97	67.74
	After Size correction	Female	< 24 months	30	0.065	1845.262	0.8178	73.33	56.67	65.00	62.86	68.00
			> 24 months	30	0.058	4189.134	0.6261	80.00	83.33	81.67	82.76	80.65
		Male	< 24 months	30	0.069	613.373	0.9774	50.00	63.33	56.67	57.69	55.88
			> 24 months	30	0.047	3119.043	0.6967	73.33	73.33	73.33	73.33	73.33

Procrustes distance (PrD), Hotelling t^2 statistic (t^2). Significance level: (*) $p < 0.05$; (**) $p<0.001$. Optimal nutrition condition (ONC), severe acute malnutrition (SAM). Sensibility (Se) and specificity (Sp) estimated for SAM.
